# The Function of *SD1* on Shoot Length and its Pyramiding Effect on Shoot Length and Plant Height in Rice (*Oryza sativa* L.)

**DOI:** 10.1186/s12284-024-00699-8

**Published:** 2024-03-25

**Authors:** Jingfang Dong, Yamei Ma, Haifei Hu, Jian Wang, Wu Yang, Hua Fu, Longting Zhang, Jiansong Chen, Lian Zhou, Wenhui Li, Shuai Nie, Ziqiang Liu, Junliang Zhao, Bin Liu, Tifeng Yang, Shaohong Zhang

**Affiliations:** 1 Rice Research Institute, Guangdong Key Laboratory of New Technology in Rice Breeding, Rice Engineering Laboratory, Key Laboratory of Genetics and Breeding of High -Quality Rice in Southern China (Co-construction by Ministry and Province), Ministry of Agriculture and Rural Affairs, Guangdong Academy of Agricultural Sciences, 510640 Guangzhou, Guangdong China; 2 College of Agriculture, South China Agricultural University, 510642 Guangzhou, China

**Keywords:** Shoot Length, Plant Height, Causal gene, Allele Mining, Pyramiding Effect, Rice

## Abstract

Strong seedling vigor is imperative to achieve stable seedling establishment and enhance the competitiveness against weeds in rice direct seeding. Shoot length (SL) is one of the important traits associated with seedling vigor in rice, but few genes for SL have been cloned so far. In the previous study, we identified two tightly linked and stably expressed QTLs for SL, *qSL-1f* and *qSL-1d* by genome-wide association study, and cloned the causal gene (*LOC_Os01g68500*) underlying *qSL-1f*. In the present study, we identify *LOC_Os01g66100* (i.e. the semidwarf gene *SD1*), a well-known gene controlling plant height (PH) at the adult-plant stage, as the causal gene underlying *qSL-1d* through gene-based haplotype analysis and knockout transgenic verification. By measuring the phenotypes (SL and PH) of various haplotypes of the two genes and their knockout lines, we found *SD1* and *LOC_ Os01g68500* controlled both SL and PH, and worked in the same direction, which provided the directly genetic evidence for a positive correlation between SL and PH combined with the analysis of SL and PH in the diverse natural population. Moreover, the knockout transgenic experiments suggested that *SD1* had a greater effect on PH compared with *LOC_ Os01g68500*, but no significant difference in the effect on SL. Further investigation of the pyramiding effects of *SD1* and *LOC_Os01g68500* based on their haplotype combinations suggested that *SD1* may play a dominant role in controlling SL and PH when the two genes coexist. In this study, the effect of *SD1* on SL at the seedling stage is validated. In total, two causal genes, *SD1* and *LOC_ Os01g68500*, for SL are cloned in our studies, which controlled both SL and PH, and the suitable haplotypes of *SD1* and *LOC_ Os01g68500* are beneficial to achieve the desired SL and PH in different rice breeding objectives. These results provide a new clue to develop rice varieties for direct seeding and provide new genetic resources for molecular breeding of rice with suitable PH and strong seedling vigor.

**Supplementary Information:**

The online version contains supplementary material available at 10.1186/s12284-024-00699-8.

## Background

Rice (*Oryza sativa* L.) is a major food crop for more than half of the world’s population (Zeng et al. 2017), and safe production is tremendously significant for world food security. Traditional rice cultivation is generally performed via puddle transplanting, which not only provides suitable soil conditions for seedling rooting and survival, but also provides reasonably good weed control (Singh et al. 2001). However, with the reduction of rural labor and the development of mechanized agriculture, people are more and more inclined to direct seed rice, a simple, labor-saving and efficient cultivation technique (Farooq et al. 2011). Previous studies have shown direct seeding can reduce total labor requirements by 11–66% depending on the season, location and type of direct seeded rice, and significantly improve production efficiency compared to traditional puddle transplanting (Kumar and Ladha 2011; Chakraborty et al. 2017).

Although direct seeding rice has many advantages, it still faces severe problems and challenges in application and promotion because modern varieties were selected through traditional transplanting methods thus, are not adapted to direct seeding conditions. Many modern rice varieties are prone to problems such as poor seedling emergence and serious weed infestation in a direct seeding system (Li et al. 2023). Therefore, it is necessary to develop rice varieties with strong seedling vigor, which is an imperative trait for stable seedling establishment and enhancing weed competitiveness in rice direct seeding system (Zhang et al. 2005; Mahender et al. 2015). However, seedling vigor is a complex trait controlled by multiple genes (Zhang et al. 2005; Sandhu et al. 2015; Yang et al. 2021, 2023; Zeng et al. 2021), thus, it is difficult to develop rice varieties with strong seedling vigor using the conventional breeding methods. Understanding its genetic basis and carrying out molecular breeding is an efficient and effective way to develop rice varieties with strong seedling vigor.

Shoot length (SL)/seedling height is one of the important traits associated with seedling vigor in rice (Lu et al. 2016). Varieties with long SL can not only enhance seedling emergence, but also improve their advantage in competition of nutrient and light energy, and effectively suppress the growth of weeds (Abe et al. 2012; Lu et al. 2016; Rao et al. 2007; Singh et al. 2017; Dimaano et al. 2020). To date, more than 100 QTLs for seedling vigor in rice have been identified by QTL analysis in bi-parental populations (Redona et al. 1996; Zhang et al. 2005; Lu et al. 2007; Zhou et al. 2007; Cairns et al. 2009; Abe et al. 2012; Yano et al. 2012; Diwan et al. 2013; Sandhu et al. 2015; Cordero-Lara et al. 2016; Singh et al. 2017; Zhang et al. 2017; Dimaano et al. 2020; Yang et al. 2021) and genome-wide association study (GWAS) in diverse natural populations (Dang et al. 2014; Anandan et al. 2016; Lu et al. 2016; Chen et al. 2019; Zhao et al. 2019; Zeng et al. 2021; Ma et al. 2022; Yang et al. 2023). However, most of the reported QTLs were identified in a single environment, and their reliability and stability remain unclear, and more importantly, few of their functional genes have been identified except *OsGA20ox1* (Abe et al. 2012; Yano et al. 2012) and *LOC_Os01g68500* (Yang et al. 2023).

Plant architecture is crucial to crop yield, and modification of rice plant type led to a dramatic increase in grain yield, among which plant height (PH) is a pivotal factor affecting plant type and straw biomass, thus considered an important agronomic trait contributing to rice yield (Khush 2003; Wang and Li 2008). Deployment of the rice semi-dwarf gene (*sd1*) triggered the “Green Revolution” in agriculture which dramatically elevated rice yields and subsequently fed a significant proportion of the global population (Evans 1998; Hedden 2003). At present, *sd1* remains one of the most important genes widely deployed in the world because of its rich polymorphic nature (Spielmeyer et al. 2002; Asano et al. 2007; Peng et al. 2021). Unfortunately, utilization of *sd1* requires heavy nitrogen fertilization to achieve high yields, and this yield advantage attributed to *sd1* has reached a bottleneck (Cheng et al. 2022) because the grain yield of semi-dwarf varieties was increased at the expense of straw biomass. Also, the direct genetic evidence of a correlation between PH and SL had not been reported.

In our previous study, two tightly linked and stably expressed QTLs for SL, *qSL-1d* and *qSL-1f* were identified in a diverse population by GWAS, and the causal gene underlying *qSL-1f*, *LOC_Os01g68500* was identified through gene-based haplotype analysis, gene expression and knockout transgenic verification (Yang et al. 2023). In this study, we further identified *LOC_Os01g66100* as the causal gene underlying *qSL-1d*, which controlled SL at the seedling stage. *LOC_Os01g66100* is identical to *SD1*, a major gene controlling PH at the adult-plant stage in rice, but its effect on SL at the seedling stage is rarely validated (Yano et al. 2012). Through measuring the phenotypes of various haplotypes of *SD1* and *LOC_Os01g68500* and their knockout lines, we discovered the two genes controlled both rice SL and PH in the same effect direction, and *SD1* had a greater effect on PH compared with *LOC_Os01g68500*, but no significant difference on SL between the two genes. Furthermore, investigating the pyramiding effects of *SD1* and *LOC_Os01g68500* on SL and PH suggested that *SD1* plays a dominant role when the two genes coexist. These results provide direct genetic evidence for the positive correlation between SL and PH, a new clue for developing direct seeded rice varieties, and new genetic resources for molecular breeding rice with suitable PH and strong seedling vigor.

## Results

### Candidate Genes Analysis of ***qSL-1d***

In our previous study, *qSL-1d* could be stably identified across multiple environments and exhibited potential value in improving SL (Yang et al. 2023). To accurately obtain the favorable haplotypes for the desired SL, it is essential to identify the causal gene underlying *qSL-1d*.

**Fig. 1:**
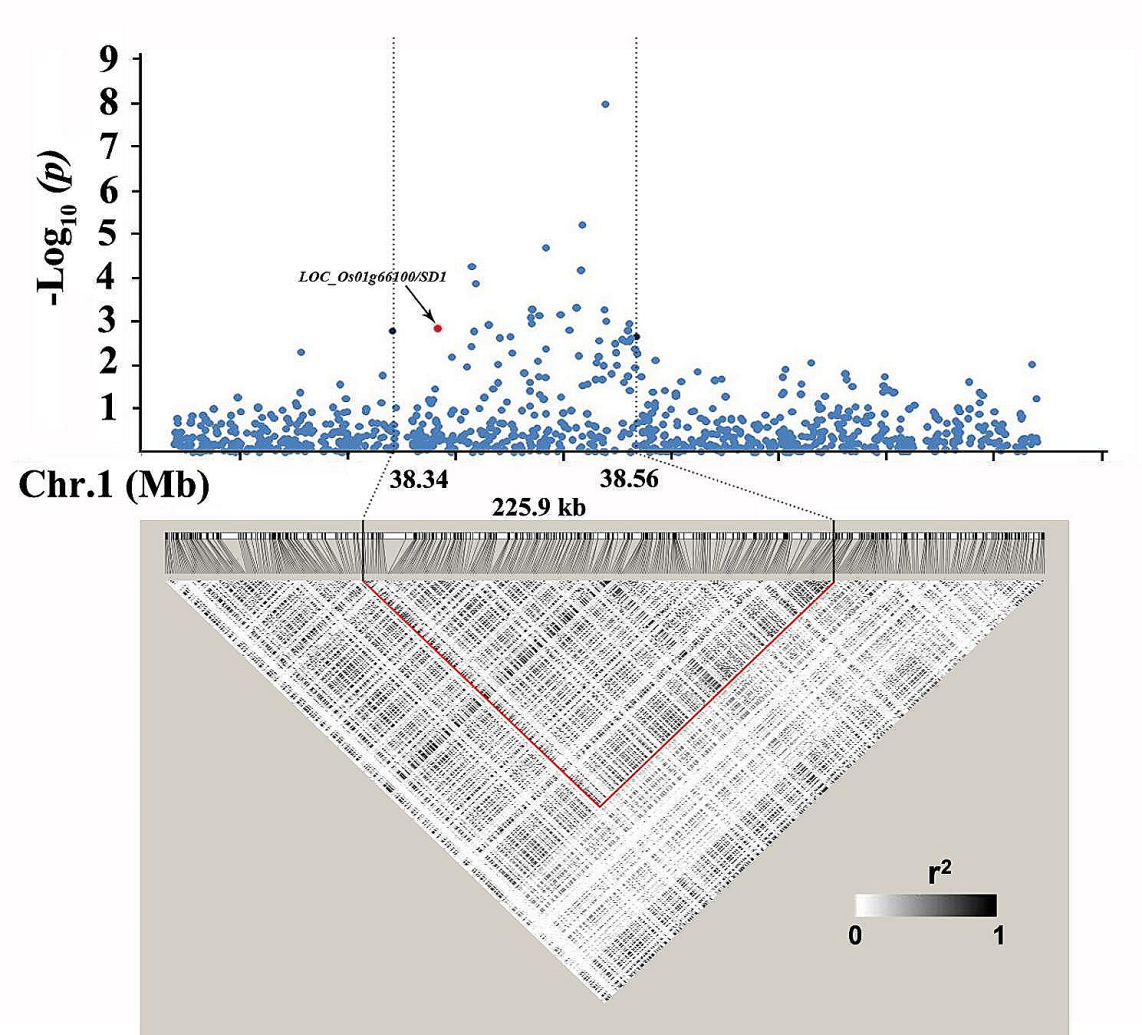
Candidate region of *qSL-1d* on chromosome 1. Local Manhattan plot (top) and LD heat map (bottom) of *qSL-1d*, indicating the candidate region between 38.34 and 38.56 Mb. The region surrounded by red triangle indicates the LD block

Analysis of linkage disequilibrium (LD) decay in the QTL region indicated that a region of approximately 225.9 kb at the associated locus was the putative region for *qSL-1d* (Fig. 1). Based on release of the MSU Rice Genome Annotation Project on the rice IRGSP-1.0 genome (http://rice.plantbiology.msu.edu/) (Kawahara et al. 2013), there are 36 annotated genes within the region of *qSL-1d*.

Using the genome re-sequencing information (50x) of 343 rice accessions from the GWAS population, the haplotypes of all annotated genes within the *qSL-1d* interval were analyzed according to the variations of each gene, and significance differences between the means of the SL for the major haplotypes (containing more than 10 lines) were tested.

**Fig. 2:**
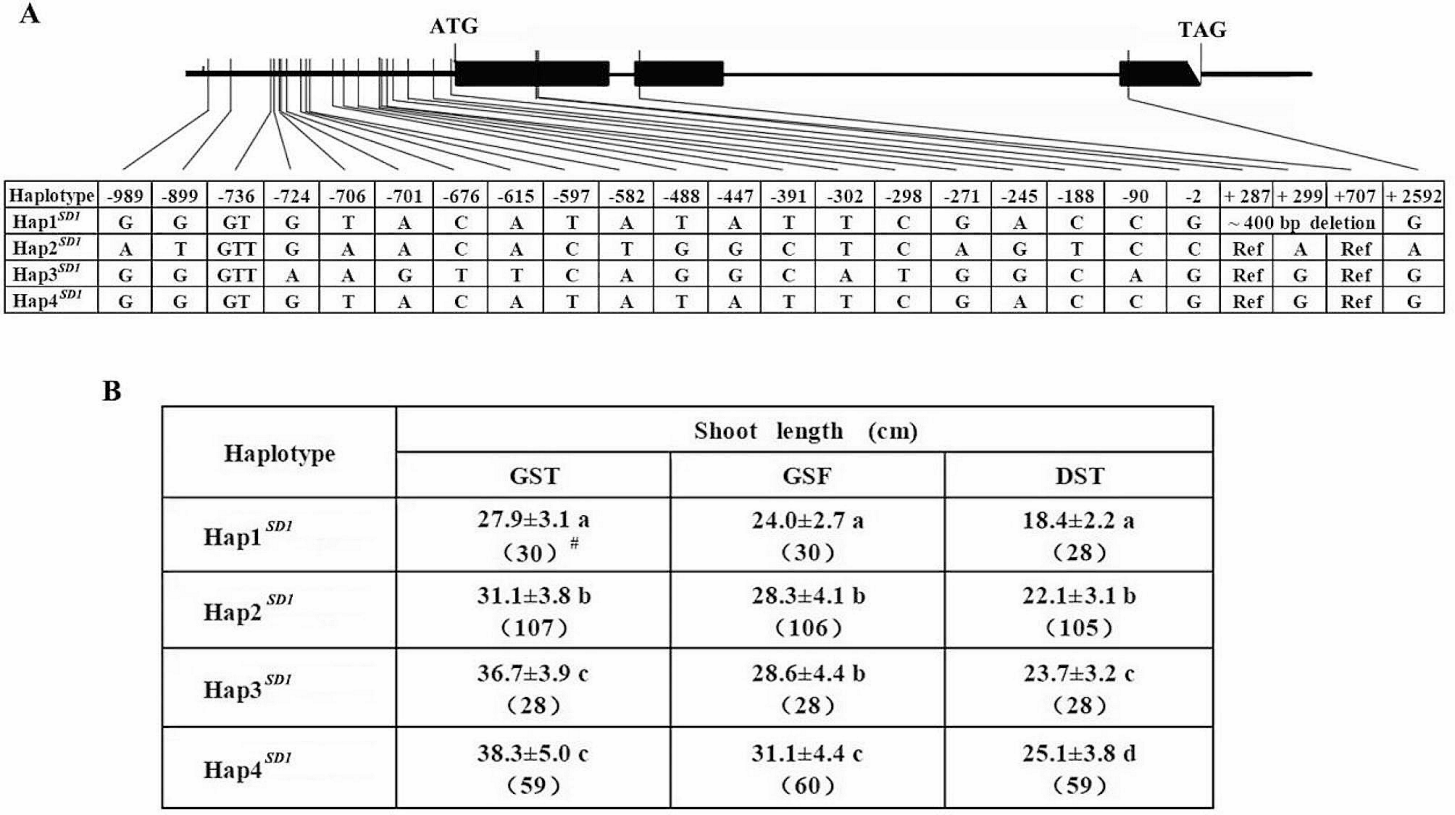
Gene structure and haplotype analysis of *SD1/LOC_Os01g66100*. **A**, The sequence variation and the resulting haplotypes of *SD1/LOC_Os01g66100*; **B**, The shoot length of various haplotypes for *SD1/LOC_Os01g66100* under GST, GSF and DST. Shoot length is presented in mean ± SD. The values with a different letter indicate a significant difference in shoot length at *P* = 0.05 based on Duncan’s multiple range test. ^#^ Numbers in parenthesis indicate the number of rice accessions with the given haplotype

Among the 36 annotated genes, only one gene, *LOC_Os01g66100* (i.e. *SD1*), was significantly different for SL among the haplotypes (Fig. 2, Table S2). There was one InDel and nineteen SNPs in the promoter region (1 kb upstream of the predicted transcription initiation site), with one structural variant and two SNPs in the coding DNA sequence (CDS) region. Four main haplotypes were identified based on the variations, among which Hap2^*SD1*^ corresponds to the reference genome Nipponbare (Fig. 2A). Comparing their sequences, Hap4^*SD1*^ and Hap1^*SD1*^ were identical in the promoter region but different in the CDS region, while Hap4^*SD1*^ and Hap3^*SD1*^ were different in the promoter region but identical in the CDS region. In the CDS region, there were only about 400-bp deletion difference between Hap1^*SD1*^ and Hap3^*SD1*^ /Hap4^*SD1*^, which occurred from the middle of exon 1 (+ 287) to upstream of exon 2 (+ 707) in Hap1^*SD1*^ and resulting in frame shift; while there were two SNPs (A-to-G transition) at the position of + 299 in exon 1 and at + 2592 in exon 3 between Hap2^*SD1*^ and Hap3^*SD1*^*/* Hap4^*SD1*^, resulting in an amino acid substitution from glutamic acid to glycine and from glutamine to arginine, respectively. Phenotypic evaluation revealed significant difference in SL among the four haplotypes (*P* < 0.05), the lines carrying Hap1^*SD1*^ exhibited the shortest SL, while the lines carrying Hap4^*SD1*^ exhibited the longest SL under the three cultivation methods (Fig. 2B), suggesting that *SD1* may be the candidate gene underlying *qSL-1d*.

### *SD1* is the Causal Gene for Shoot Length of Seedling

**Fig. 3:**
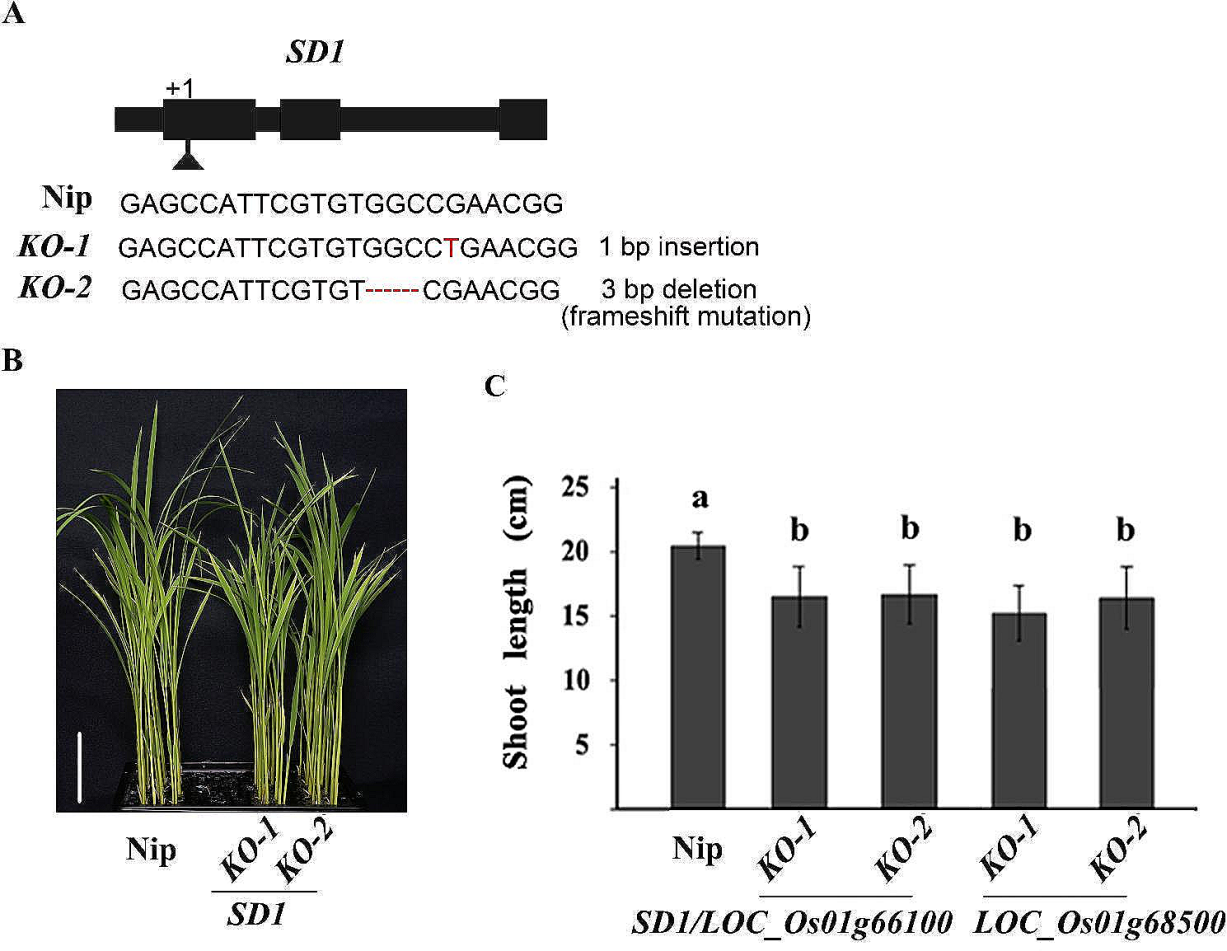
Mutation types and shoot length of the knockout transgenic lines of *SD1/LOC_Os01g66100* (in the present study) and *LOC_Os01g68500* (Yang et al. 2023). **A**, The mutation types of *SD1/LOC_Os01g66100*; **B**, Shoot length of wild-type Nipponbare (Nip) and the knockout transgenic (KO) lines of *SD1/LOC_Os01g66100*. Scale bar is 3 cm; **C**, Multiple comparisons of shoot length among wild-type Nipponbare (Nip), the knockout transgenic (KO) lines of *SD1/LOC_Os01g66100* and *LOC_Os01g68500*. The different letters above the histogram indicate the significant difference at *P* = 0.05 based on Duncan’s multiple range test

To validate the effect of *SD1* on SL, CRISPR/Cas9 was applied to knock out *SD1* in Nipponbare and *SD1* knockout transgenic (KO) lines were constructed. In T_2_ generation, we selected two homozygous lines with complete mutation, *KO-1* and *KO-2*, which caused frameshift mutations due to 1 bp insertion and 3 bp deletion, respectively, for SL measurement (Fig. 3A). The results showed that the SL in Nipponbare (the wild type) was 20.5 cm, while in *KO-1* and *KO-2* was 16.6 and 16.8 cm, respectively, with an average of 18.5% SL reduction in the KO lines compared to their wild type (Fig. 3B-C). The SLs of the mutants were significantly shorter than that of their wild type (*P* < 0.05), indicating that *SD1* is the causal gene for SL in rice. Comparing the effects of *SD1* (this study) and *LOC_Os01g68500* (Yang et al. 2023) on SL, no significant difference was found (Fig. 3C).

### The Effects of ***SD1*** (***LOC_Os01g66100***) and ***LOC_Os01g68500*** on Plant Height of Adult Plant

*SD1* (*LOC_Os01g66100*) controlled not only the SL of seedling (the present study), but also the PH of adult plant (Sasaki et al. 2002). *LOC_Os01g68500* is a newly reported functional gene for SL (Yang et al. 2023), but its role in PH remains unclear.

**Fig. 4:**
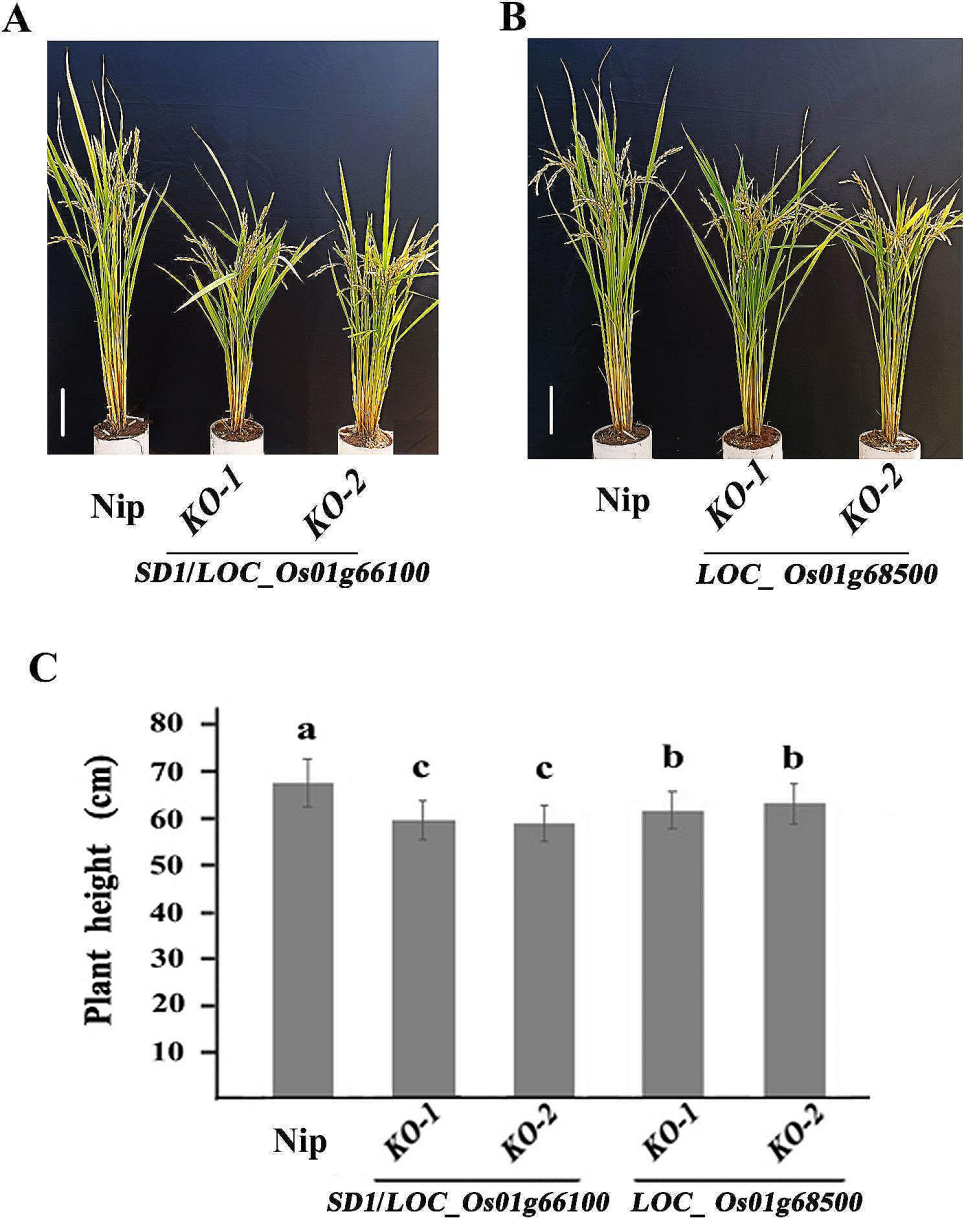
The plant height of the knockout transgenic lines of *SD1/LOC_Os01g66100* and *LOC_Os01g68500*. **A** and **B**, Plant height of wild-type Nipponbare (Nip) and the knockout transgenic (KO) lines of *SD1/LOC_Os01g66100* (A) and *LOC_Os01g68500* (B). Scale bar is 10 cm; **C**, Comparisons of plant height among wild-type Nipponbare (Nip) and the knockout transgenic (KO) lines of *SD1/LOC_Os01g66100* and *LOC_Os01g68500*. The different letters above the histogram indicate the significant difference at *P* = 0.05 based on Duncan’s multiple range test

We first measured the PH in the KO lines of the two genes. The results exhibited that their KO lines also significantly reduced the PH (*P* < 0.05), with a 13.1% and 7.6% reduction in PH of the KO lines of *SD1* and *LOC_Os01g68500*, respectively, compared with their wild type (Fig. 4A-C), suggesting that *LOC_Os01g68500* also controls PH as same as *SD1* does, but *SD1* had a greater effect on PH compared with *LOC_Os01g68500*.

In order to verify the biological functions of the two genes on PH and identify different alleles affecting PH, we further analyzed the PH based on their haplotypes in the GWAS population. The results showed that there were significant differences in PH among the four haplotypes of *SD1*, with the shortest in Hap1^*SD1*^ and the tallest in Hap3^*SD1*^ and Hap4^*SD1*^ (Table 1), which is consistent with the trend in SL (Fig. 2B). For *LOC_Os01g68500*, which contained two main haplotypes identified in our previous study (Yang et al. 2023), the PH of Hap1^*LOC_Os01g68500*^ was significantly shorter than that of Hap2^*LOC_Os01g68500*^ (Table 1), which is also consistent with the trend in SL (Yang et al. 2023). These results suggested that both variations in SL at seedling stage and PH at adult stage were controlled by the same alleles/haplotypes of these two genes.

**Table 1:** The plant height of various haplotypes of *SD1/LOC_Os01g66100* and *LOC_Os01g68500*

Genes	Haplotype	Plant height (cm)	
		Guangzhou	Yangjiang
*SD1*			
*/LOC_Os01g66100*			
	Hap1^*SD1*^	91.8 ± 10.1 a (25)	90.1 ± 8.9 a (26)
	Hap2^*SD1*^	120.7 ± 24.2 b (91)	125.1 ± 19.1 b (92)
	Hap3^*SD1*^	136.8 ± 18.2 c (28)	142.9 ± 19.2 c (28)
	Hap4^*SD1*^	137.2 ± 21.3 c (46)	142.9 ± 18.0 c (56)
*LOC_Os01g68500*			
	Hap1^*LOC_Os01g68500*^	122.6 ± 26.7 ** (174)	124.8 ± 24.0 ** (154)
	Hap2^*LOC_Os01g68500*^	141.0 ± 17.8 (88)	143.4 ± 17.0 (85)

Plant height is presented in mean ± SD. The values with different letter indicate significant difference in plant height at *P* = 0.05 based on Duncan’s multiple range test; ** indicates the significant difference at *P* = 0.01 based on *t*-test; Numbers in parenthesis indicate the number of rice accessions

### The Correlation between Shoot Length at Seedling Stage and Plant Height at Adult Stage

*SD1* and *LOC_ Os01g68500* were identified to be the functional genes controlling SL and PH in the same effect direction (Figs. 2B, 3 and 4; Table 1 and Yang et al. 2023), suggesting a correlation between SL and PH. Given this relationship, we analyzed the correlations between SL and PH in the same GWAS population. The results showed a significant positive correlation (*P*<0.01), with correlation coefficients of 0.481/0.540, 0.339/0.448 and 0.512/0.510 between PH in Guangzhou/Yangjiang and SL under the cultivation method of GST, GSF and DST, respectively (Table 2).

**Table 2:** The pairwise correlation coefficients between shoot length using the three cultivation methods and plant height at the two locations

	Shoot length (GST)	Shoot length (GSF)	Shoot length (DST)	Plant height (Guangzhou)	Plant height (Yangjiang)
Shoot length (GST)	1.000				
Shoot length (GSF)	0.726**	1.000			
Shoot length (DST)	0.769**	0.787**	1.000		
Plant height (Guangzhou)	0.481**	0.339**	0.512**	1.000	
Plant height (Yangjiang)	0.540**	0.448**	0.510**	0.927**	1.000

** Significance level at *P* < 0.01

### The Pyramiding Effects of ***SD1*** and ***LOC_Os01g68500*** on Shoot Length and Plant Height

Pyramiding the suitable alleles/haplotypes for target traits is very important for developing rice varieties with the desired performance for different breeding objectives. Being the functional genes of the two stably expressed QTLs which would be beneficial to improve both SL and PH, the pyramiding effects of the two genes were investigated based on their haplotypes.

For *SD1*, four main haplotypes were identified in this study, of which Hap3^*SD1*^ and Hap4^*SD1*^ exhibited longer SL and taller PH than Hap1^*SD1*^ and Hap2^*SD1*^ (Fig. 2; Table 1); for *LOC_Os01g68500*, two major haplotypes were identified based on a single base variation in its CDS region, of which Hap2^*LOC_Os01g68500*^ exhibited longer SL and taller PH than Hap1^*LOC_Os01g68500*^ (Yang et al. 2023 and Table 1). To analyze the pyramiding effects of the two genes, the rice accessions in the GWAS population were grouped according to their haplotypes and six haplotype combinations were found (Table 3). Among the six haplotype combinations, combination 1 (Hap1^*SD1*^ *+* Hap1^*LOC_Os01g68500*^) exhibited the shortest SL and PH, while both combination 5 (Hap4^*SD1*^ *+* Hap1^*LOC_Os01g68500*^) and combination 6 (Hap4^*SD1*^ *+* Hap2^*LOC_Os01g68500*^) exhibited the longest SL and the tallest PH, which seems to be consistent with the presence of the single gene, i.e., Hap1^*SD1*^ or Hap1^*LOC_Os01g68500*^ exhibited lower SL and PH, while Hap4^*SD1* or^ Hap2^*LOC_Os01g68500*^ exhibited longer SL and taller PH (Yang et al. 2023, Fig. 2; Table 1). However, comparison of the haplotype combinations showed that there were significant differences in SL and PH between combination 1 (Hap1^*SD1*^ *+* Hap1^*LOC_Os01g68500*^) and combination 2 (Hap2^*SD1*^ *+* Hap1^*LOC_Os01g68500*^), but no significant differences in SL and PH between combination 3 (Hap3^*SD1*^ *+* Hap1^*LOC_Os01g68500*^) and combination 4 (Hap3^*SD1*^ *+* Hap2^*LOC_Os01g68500*^), as well as between combination 5 (Hap4^*SD1*^ *+* Hap1^*LOC_Os01g68500*^) and combination 6 (Hap4^*SD1*^ *+* Hap2^*LOC_Os01g68500*^) (Table 3), suggesting that *SD1* may play a dominant role in controlling both SL and PH when the two gene coexist.

**Table 3:** The pyramiding effect of *SD1/LOC_Os01g66100* and *LOC_Os01g68500* on shoot length and plant height

Combinations	Gene			Shoot length (cm)				Plant height (cm)	
	SD1	LOC_Os01g68500		GST	GSF	DST		Guangzhou	Yangjiang
1	Hap1^*SD1*^	Hap1^*LOC_Os01g68500*^		27.7 ± 2.3 a (19)	24.0 ± 2.5 a (20)	18.6 ± 2.2 a (20)		91.6 ± 9.8 a (20)	91.9 ± 10.0 a (16)
2	Hap2^*SD1*^	Hap1^*LOC_Os01g68500*^		30.9 ± 3.7 b (74)	27.6 ± 3.3 b (74)	22.0 ± 2.7 b (74)		122.7 ± 23.6 b (74)	121.9 ± 18.0 b (67)
3	Hap3^*SD1*^	Hap1^*LOC_Os01g68500*^		36.5 ± 4.6 b (8)	27.9 ± 3.7 b (8)	22.9 ± 3.5 b (8)		130.6 ± 19.5 bc (8)	138.0 ± 20.9 c (8)
4	Hap3^*SD1*^	Hap2^*LOC_Os01g68500*^		36.3 ± 3.9 b (15)	27.4 ± 4.2 b (15)	23.3 ± 3.2 bc (15)		140.6 ± 16.9 c (15)	146.0 ± 19.1 c (15)
5	Hap4^*SD1*^	Hap1^*LOC_Os01g68500*^		40.2 ± 5.0 c (14)	31.9 ± 3.4 c (14)	25.4 ± 3.3 c (14)		141.7 ± 19.3 c (14)	141.7 ± 18.7 c (13)
6	Hap4^*SD1*^	Hap2^*LOC_Os01g68500*^		38 ± 4.9 bc (29)	31.5 ± 4.3 c (29)	25.4 ± 3.3 c (29)		134.9 ± 22.3 bc (29)	138.8 ± 20.7 c (26)

Shoot length and plant height are presented in mean ± SD. The values with different letter indicate the significant difference at *P* = 0.05 based on Duncan’s multiple range test. Numbers in parenthesis indicate the number of rice accessions

## Discussion

### *SD1* Controls Shoot Length at the Seedling Stage in Rice

Although great progress has been made in the analysis of the genetic basis of SL in rice, few of their functional genes have been identified except for *qPHS3-2* (Abe et al. 2012)/*qEPD2* (Yano et al. 2012) and *qSL-1f* (Yang et al. 2023). In this paper, we report that *LOC_Os01g66100*, which is identical to the semi-dwarf gene *SD1*, was the causal gene underlying *qSL-1d*, a stably expressed QTL for SL identified in our previous study (Yang et al. 2023).

*SD1*, a major gene controlling the PH at the adult plant stage in rice (Sasaki et al. 2002; Asano et al. 2011), encodes gibberellin 20-oxidase (GA20ox2), a key enzyme in gibberellic acid (GA) biosynthesis, catalyzing the conversion of GA53 to GA20 (Sasaki et al. 2002). Previous studies reported that *SD1* could also regulate seed dormancy (Ye et al. 2015), panicle structure (Su et al. 2021) and grain weight (Zhang et al. 2013) in rice, however, its effect on SL of seedling is rarely validated. In this study, gene knockout experiments showed an average of 18.5% SL reduction in the *SD1* KO lines compared to their wild type (Fig. 3B), indicating that *SD1* controls SL in rice.

Four major haplotypes of *SD1* were identified in the present study. The sequence comparisons revealed that there was only a deletion difference between Hap1^*SD1*^ and Hap3^*SD1*^*/*Hap4^*SD1*^ in the CDS region, while there was sequence difference between Hap3^*SD1*^ and Hap4^*SD1*^ but none between Hap1^*SD1*^ and Hap4^*SD1*^ in the promoter region. The lines carrying Hap1^*SD1*^ had a deletion of about 400-bp from the middle of exon 1 to upstream of exon 2, resulting in frame shift, and consequently a significantly shorter SL in Hap1^*SD1*^ than Hap3^*SD1*^ and Hap4^*SD1*^ (Fig. 2), speculating that this deletion causes the functional variation of the gene, which is similar to that reported in *sd1* of a semi-dwarf mutant (Sasaki et al. 2002; Spielmeyer et al. 2002). Comparison of Hap2^*SD1*^ and Hap3^*SD1*^*/*Hap4^*SD1*^ revealed two SNPs (A-to-G transition) at the position of + 299 in exon 1 and at + 2592 in exon 3, causing two amino acid substitutions from glutamic acid to glycine and from glutamine to arginine, respectively, which are consistent with the previous results (Asano et al. 2011; Zhang et al. 2020), and also resulted in a significant difference in SL among the three haplotypes in the present study (Fig. 2). These results indicated that diverse haplotypes of *SD1* contribute to the diversity of SL variation.

### *SD1* and ***LOC_ Os01g68500*** Control Shoot Length and Plant Height in the Same Effect Direction

In the present and previous studies, we cloned two functional genes, *SD1* and *LOC_ Os01g68500* for SL by GWAS analysis and functional verification (Fig. 3 and Yang et al. 2023). As *SD1* is a major gene controlling PH, we further explored the function of the two genes on PH. The results of knockout transgenic experiments and haplotype analysis showed that *SD1*and *LOC_ Os01g68500* controlled both SL and PH in the same effect direction (Figs. 2B, 3 and 4; Table 1 and Yang et al. 2023). In the GWAS population, PH was significantly positively correlated with SL (*P*<0.01) under the multiple environments (Table 2). All of these results provide direct genetic evidence for the positive correlation between SL and PH. Since the “Green Revolution”, most rice varieties have adapted to semi-dwarf architecture owing to the advantages of semi-dwarf rice varieties such as fertilizer tolerance and lodging resistance, but the reduction in PH may also result in reduced SL due to their positive correlation. More importantly, the grain yield of semi-dwarf varieties was increased at the expense of straw biomass, which would cause a yield bottleneck. Therefore, improving biomass has become a common goal in attempts to increase rice yield, especially for rice breeding programs focused on super-high yield (Ying et al. 1998; Peng et al. 1999). Based on this concept, developing rice varieties with longer SL can not only improve the seedling vigor to control weeds, but also improve biomass by increasing PH to achieve high-yielding rice. However, taller PH may induce the risk of lodging under the direct seeding condition. To address this issue, pyramiding gene(s) for lodging resistance may be effective, but introgression of the other genes is a time-consuming process. Fortunately, of the two genes identified in our studies, the effect of *SD1* on PH was stronger than that of *LOC_Os01g68500* (Fig. 4C), but no significant difference on SL between the two genes (Fig. 3C), suggested that using *LOC_Os01g68500* to develop rice varieties with longer SL would reduce the risk of lodging.

### The Suitable Haplotypes of ***SD1*** and ***LOC_Os01g68500*** are Beneficial to Achieve the Desired Shoot Length and Plant Height in Rice Breeding

The use of natural allelic variation is important for molecular design in rice breeding. In order to make better use of cloned genes for molecular breeding, it is necessary to understand their haplotypes to obtain the desired haplotype. In our studies, four and two main haplotypes for *SD1* and *LOC_Os01g68500* were identified, respectively, through gene-based haplotype analysis in 343 diverse rice accessions. Sequence analysis reveals several variations that cause the significant difference in SL and PH among the haplotypes (Fig. 2; Table 1 and Yang et al. 2023), which could be considered as a functional variant and could be used in molecular breeding. In addition, the pyramiding effects of *SD1* and *LOC_Os01g68500* showed that there was no significant difference in SL and PH between combination 3 (Hap3^*SD1*^ *+* Hap1^*LOC_Os01g68500*^) and combination 4 (Hap3^*SD1*^ *+* Hap2^*LOC_Os01g68500*^), as well as between combination 5 (Hap4^*SD1*^ *+* Hap1^*LOC_Os01g68500*^) and combination 6 (Hap4^*SD1*^ *+* Hap2^*LOC_Os01g68500*^) (Table 3), suggesting that *SD1* may play a dominant role in controlling SL and PH when the two genes coexist, or *SD1* may play its biological function at the upstream of *LOC_Os01g68500.* Of course, more robust genetic evidence for the pyramiding effect could be provided by determining SL and PH in the progeny derived from the cross between *SD1-KO* and *LOC_ Os01g68500-KO* plants. This research is ongoing in our laboratory and we will continue to elucidate the pyramiding phenotype and the deeper molecular mechanisms of these two genes.

Based on the knowledge from this study, molecular markers for different target haplotypes/alleles can be developed based on the functional variations of the two genes, and the most suitable alleles can be used to breed varieties with the desired SL and PH for different breeding objectives.

## Conclusion

In the present study, the effect of *SD1* on SL at the seedling stage is validated through gene-based haplotype analysis and knockout transgenic experiment, and its main haplotypes affecting SL were also identified. In total, two causal genes, *SD1* and *LOC_ Os01g68500*, for SL are cloned in our studies, which controlled both SL and PH and worked in the same direction. Although the pyramiding effects of *SD1* and *LOC_Os01g68500* suggested that *SD1* may play a dominant role in controlling SL and PH when the two genes coexist, the suitable haplotypes of the two genes can be used to develop varieties with the desired SL and PH for different breeding objectives.

## Materials and Methods

### Plant Materials

In our previous study (Yang et al. 2023), we selected 391 rice accessions from the 1,568 accessions included in the Rice Diversity Panel 2 (McCouch et al. 2016) based on their genetic diversity and originating country. This subset of accessions is listed in Table S1 and designated as the “GWAS population” which originated from 56 countries.

The 1,568 accessions were obtained from the International Rice Research Institute genebank in 2013. A quarantine grow-out was conducted at the Guangzhou Experimental Station in Guangdong Province and the harvested seed used for these studies. All seeds used in this study were newly increased in the experimental year. The seeds were stored at room temperature for three months after harvest, then used for phenotypic evaluation.

### Evaluation of Shoot Length at the Seedling Stage

For the GWAS population, SL evaluation was carried out using the three cultivation methods reported by Yang et al. (2023). Briefly, Method 1 (GST): seeds were pre-germinated and sown in plastic trays; Method 2 (GSF): seeds were pre-germinated and sown in the paddy field; Method 3 (DST): seeds were directly sown in plastic trays without pre-germination. GST studies were conducted in May 2018, while GSF and DST studies were conducted between late April and early May 2019. SL of seedlings were measured after 14 days of growth in natural environment and this data from Yang et al. (2023) is included in Table S1.

For SD1 knockout transgenic (KO) lines, the healthy and filled seeds of KO lines and its wild-type line were incubated at 49℃ for 96 h to break dormancy. After sterilization in 3% sodium hypochlorite solution, the seeds were soaked in distilled water for 24 h. The pre-germinated seeds were sown in black plastic culture boxes (12 cm×8.6 cm×11 cm) filled with 0.1% Yoshida nutrient solution, then put into a growth chamber set at 30℃, 70% relative humidity and a 12 h light/12 h dark cycle. After 14 days, the SL of the seedling were measured. Four replicates with 20 plants per line were used in SL evaluation.

### Evaluation of Plant Height at the Adult-Plant Stage

For haplotype analysis, the GWAS population was planted in the paddy field at the Guangzhou and Yangjiang Experimental Stations in Guangdong Province, China. The experiments were conducted in the second cropping season (July to November) in Guangzhou (2016) and Yangjiang (2018) and arranged in a completely randomized block design with two replications. The germinated seeds were sown in a seedling bed and sixteen 15-day-old seedlings were transplanted into two rows in the field with an individual plant space of 20 cm×20 cm. After maturity, the PHs of 12 individuals in the middle of rows were measured and averaged.

For knockout transgenic lines, the *SD1* and *LOC_Os01g68500* KO lines and their wild-type line were planted in the transgenic experimental field in the second cropping season (July to November) at Guangzhou (2022) and arranged in a completely randomized block design with three replications. Twenty-four 15-day-old seedlings were transplanted into three rows in the field with an individual plant space of 20 cm×20 cm. After maturity, the PHs of the individuals in the middle of rows were measured.

The field management, including irrigation, fertilization, and disease and pest control, followed the conventional practice for rice production.

### DNA Sequence Analysis

Only 343 accessions in the GWAS population (identified in Table S1) were re-sequenced using the Illumina NovaSeq6000 platform, and the details of sequencing data analysis were described in our previous study (Wang et al. 2023; Yang et al. 2023). All raw sequence data have been deposited in the NCBI sequence read archive (BioProject accession PRJNA820969).

### Gene Haplotype Analysis and Candidate Gene Identification

Gene haplotype analysis and candidate gene identification were carried out as described in our previous study (Yang et al. 2023). First, the indel, SNP and PAV (the presence/absence variation > 50 bp) within the QTL interval were analyzed with Nipponbare as the reference genome using the re-sequencing information (50×) for the 343 rice accessions. Next, all annotated genes within the QTL interval were examined to identify their haplotypes based on their sequence variations. Then the accessions were grouped based on the haplotypes of each gene and the post hoc multiple comparison with Duncan function was performed to identify the significant differences in SL among the major haplotypes (containing more than 10 accessions). A gene was considered a candidate gene if the significant differences in SL were observed among haplotypes of a gene under all cultivation environments.

### Validation of Candidate Gene for Shoot Length

In order to validate the function of *SD1* on SL, we conducted the knockout transgenic experiments. To generate the CRISPR/Cas9 vectors, *SD1* single guide RNA (sgRNA) sequences were cloned using pYLgRNA-OsU3 as described previously (Ma et al. 2015). The target site sequence of *SD1* was 5’-CCGTTCGGCCACACGAATGGCTC-3’, which contained a protospacer adjacent motif (PAM) CGG at the 3’ end. The positive plasmids were electroporated into *Agrobacterium tumefaciens* EHA105, then introduced into calli of the cultivar Nipponbare via Agrobacterium-mediated genetic transformation.

At the T_2_ generation, the homozygous positive transgenic plants of *SD1* were selected by gene cloning and sequencing. The seeds of the homozygous positive plants were used to evaluate SL and PH, and the wild-type plants (Nipponbare) were used as control.

The knockout transgenic lines of *LOC_Os01g68500* were developed in our previous study (Yang et al. 2023).

### Data Analysis

A *t*-test or Duncan’s multiple range test was conducted using *SPSS*10.0 to detect the differences in SL and PH between or among the tested rice accessions.

### Electronic Supplementary Material

Below is the link to the electronic supplementary material.

Supplementary Material 1

## Data Availability

No datasets were generated or analysed during the current study.

## Abbreviations

CDS: Coding DNA Sequence
DST: Direct seeding into the trays
GSF: Pre-germinated seeds were sown in field
GST: Pre-germinated seeds were sown in trays
GWAS: Genome-wide association study
LD: Linkage disequilibrium
PH: Plant height
QTLs: Quantitative trait loci
SL: Shoot length
SNP: Single nucleotide polymorphism

## References

[CR1] Abe A, Takagi H, Fujibe T, Aya K, Kojima M, Sakakibara H, Uemura A, Matsuoka M, Terauchi R (2012). *OsGA20ox1*, a candidate gene for a major QTL controlling seedling vigor in rice. Theor Appl Genet.

[CR2] Anandan A, Anumalla M, Pradhan SK, Ali J (2016). Population structure, diversity and trait association analysis in rice (*Oryza sativa* L.) germplasm for early seedling vigor (ESV) using trait linked SSR markers. PLoS ONE.

[CR3] Asano K, Takashi T, Miura K, Qian Q, Kitano H, Matsuoka M, Ashikari M (2007). Genetic and molecular analysis of utility of *SD1* alleles in rice breeding. Breed Sci.

[CR4] Asano K, Yamasaki M, Takuno S, Miura K, Katagiri S, Ito T, Doi K, Wu JZ, Ebana K, Matsumoto T, Innan H, Kitano H, Ashikari M, Matsuoka M (2011). Artificial selection for a green revolution gene during japonica rice domestication. Proc Natl Acad Sci USA.

[CR5] Cairns JE, Namuco OS, Torres R, Simborio FA, Courtois B, Aquino GA, Johnson DE (2009). Investigating early vigour in upland rice (*Oryza sativa* L.): part II. Identification of QTLs controlling early vigour under greenhouse and field conditions. Field Crop Res.

[CR6] Chakraborty D, Ladha JK, Rana DS, Jat ML, Gathala MK, Yadav S, Rao AN, Ramesha MS, Raman A (2017). A global analysis of alternative tillage and crop establishment practices for economically and environmentally efficient rice production. Sci Rep.

[CR7] Chen K, Zhang Q, Wang C, Liu Z, Jiang Y, Zhai L, Zheng T, Xu J, Li Z (2019). Genetic dissection of seedling vigour in a diverse panel from the 3,000 Rice (*Oryza sativa* L.) Genome Project. Sci Rep.

[CR8] Cheng X, Huang Y, Tan Y, Tan L, Yin J, Zou G (2022). Potentially useful dwarfing or semi-dwarfing genes in rice breeding in addition to the *SD1* gene. Rice (N Y).

[CR9] Cordero-Lara K, Kim H, Tai T (2016). Identification of seedling vigor-associated quantitative trait loci in temperate japonica rice. Plant Breed Biotech.

[CR10] Dang X, Thi T, Dong G, Wang H, Edzesi W, Hong D (2014). Genetic diversity and association mapping of seed vigor in rice (*Oryza sativa* L). Planta.

[CR11] Dimaano N, Ali J, Mahender A, Cruz P, Baltazar A, Diaz M, Pang Y, Li B (2020). Identification of quantitative trait loci governing early germination and seedling vigor traits related to weed competitive ability in rice. Euphytica.

[CR12] Diwan J, Channbyregowda M, Shenoy V, Salimath P, Bhat R (2013). Molecular mapping of early vigour related QTLs in rice. Res J Biology.

[CR13] Evans L (1998). Feeding the ten billion. Plant and population growth.

[CR14] Farooq M, Siddique K, Rehman H, Aziz T, Lee D, Wahid A (2011). Rice direct seeding: experiences, challenges and opportunities. Soil Tillage Res.

[CR15] Hedden P (2003). Constructing dwarf rice. Nat Biotechnol.

[CR16] Kawahara Y, Bastide M, Hamilton J, Kanamori H, McCombie WR, Ouyang S, Schwartz DC, Tanaka T, Wu J, Zhou S, Childs KL, Davidson RM, Lin H, Quesada-Ocampo L, Vaillancourt B, Sakai H, Lee SS, Kim JS, Numa H, Itoh T, Buell C, Matsumoto T (2013). Improvement of the *Oryza sativa* nipponbare reference genome using next generation sequence and optical map data. Rice (NY).

[CR18] Khush GS (2003). Productivity improvements in rice. NUTR REV.

[CR17] Kumar V, Ladha JK (2011). Direct seeding of rice: recent developments and future research needs. Adv Agron.

[CR19] Li X, Dong J, Zhu W, Zhao J, Zhou L (2023). Progress in the study of functional genes related to direct seeding of rice. Mol Breed.

[CR21] Lu X, Niu A, Cai H, Yong Z, Liu J, Zhu Y, Zhang Z (2007). Genetic dissection of seedling and early vigor in a recombinant inbred line population of rice. Plant Sci.

[CR20] Lu Q, Zhang M, Niu X, Wang C, Xu Q, Feng Y, Wang S, Yuan X, Yu H, Wang Y, Wei X (2016). Uncovering novel loci for mesocotyl elongation and shoot length in indica rice through genome-wide association mapping. Planta.

[CR22] Ma X, Zhang Q, Zhu Q, Liu W, Chen Y, Qiu R, Wang B, Yang Z, Li H, Lin Y, Xie Y, Shen R, Chen S, Wang Z, Chen Y, Guo J, Chen L, Zhao X, Dong Z, Liu Y (2015). A robust CRISPR/Cas9 system for convenient, high-efficiency multiplex genome editing in monocot and dicot plants. Mol Plant.

[CR23] Ma Y, Wang J, Yang T, Dong J, Yang W, Chen L, Zhou L, Chen J, Liu B, Zhang S, Edwards D, Zhao J (2022). Genome-wide association mapping and gene expression analysis identify *OsCPS1* as a new candidate gene controlling early seedling length in rice. Front Plant sci.

[CR25] Mahender A, Anandan A, Pradhan SK (2015). Early seedling vigour, an imperative trait for direct-seeded rice: an overview on physio-morphological parameters and molecular markers. Planta.

[CR24] McCouch S, Wright M, Tung C, Maron L, McNally K, Fitzgerald M, Singh N, DeClerck G, Agosto-Perez F, Korniliev P, Greenberg A, Naredo M, Mercado S, Harrington S, Shi Y, Branchini D, Kuser-Falcão P, Leung H, Ebana K, Yano M, Eizenga G, McClung A, Mezey J (2016). Open access resources for genome-wide association mapping in rice. Nat Commun.

[CR26] Peng S, Cassman KG, Virmani SS, Sheehy J, Khush GS (1999). Yield potential trends of tropical rice since the release of IR8 and the challenge of increasing rice yield potential. Crop Sci.

[CR27] Peng Y, Hu Y, Qian Q, Ren D (2021). Progress and prospect of breeding utilization of green revolution gene *SD1* in rice. Agriculture.

[CR28] Rao AN, Johnson DE, Sivaprasad B, Ladha JK, Mortimer AM (2007). Weed management in direct-seeded rice. Adv Agron.

[CR29] Redona ED, Mackill DJ (1996). Mapping quantitative trait loci for seedling vigor in rice using RFLPs. Theor Appl Genet.

[CR30] Sandhu N, Torres RO, Sta Cruz MT, Maturan PC, Jain R, Kumar A, Henry A (2015). Traits and QTLs for development of dry direct-seeded rainfed rice varieties. J Exp Bot.

[CR31] Sasaki A, Ashikari M, Ueguchi-Tanaka M, Itoh H, Nishimura A, Swapan D, Ishiyama K, Saito T, Kobayashi M, Khush GS, Kitano H, Matsuoka M (2002). A mutant gibberellin-synthesis gene in rice. Nature.

[CR32] Singh S, Sharma SN, Prasad R (2001). The efect of seeding and tillage methods on productivity of rice-wheat cropping system. Soil Tillage Res.

[CR33] Singh UM, Yadav S, Dixit S, Ramayya PJ, Devi MN, Raman KA, Kumar A (2017). QTL hotspots for early vigor and related traits under dry direct-seeded system in rice (*Oryza sativa* L). Front Plant sci.

[CR34] Spielmeyer W, Ellis MH, Chandler PM (2002). Semidwarf (*sd-1*), green revolution rice, contains a defective gibberellin 20-oxidase gene. PNAS.

[CR35] Su S, Hong J, Chen X, Zhang C, Chen M, Luo Z, Chang S, Bai S, Liang W, Liu Q, Zhang D (2021). Gibberellins orchestrate panicle architecture mediated by DELLA-KNOX signalling in rice. Plant Biotechnol J.

[CR36] Wang Y, Li J (2008). Molecular basis of plant architecture. Annu Rev Plant Biol.

[CR37] Wang J, Yang W, Zhang S, Hu H, Yuan Y, Dong J, Chen L, Ma Y, Yang T, Zhou L, Chen J, Liu B, Li C, Edwards D, Zhao J (2023). A pangenome analysis pipeline provides insights into functional gene identification in rice. Genome Biol.

[CR38] Yang J, Guo Z, Luo L, Gao Q, Xiao W, Wang J, Wang H, Chen Z, Guo T (2021). Identification of QTL and candidate genes involved in early seedling growth in rice via high-density genetic mapping and RNA-seq. Crop J.

[CR39] Yang T, Dong J, Zhao J, Zhang L, Zhou L, Yang W, Ma Y, Wang J, Fu H, Chen J, Li W, Hu H, Jiang X, Liu Z, Liu B, Zhang S (2023). Genome-wide association mapping combined with gene-based haplotype analysis identify a novel gene for shoot length in rice (*Oryza sativa* L). Theor Appl Genet.

[CR40] Yano K, Takashi T, Nagamatsu S, Kojima M, Sakakibara H, Kitano H, Matsuoka M, Aya K (2012). Efficacy of microarray profiling data combined with QTL mapping for the identification of a QTL gene controlling the initial growth rate in rice. Plant Cell Physiol.

[CR41] Ye H, Feng J, Zhang L, Zhang J, Mispan MS, Cao Z, Beighley D, Yang J, Gu X (2015). Map-based cloning of seed dormancy1-2 identified a gibberellin synthesis gene regulating the development of endosperm-imposed dormancy in rice. Plant Physiol.

[CR42] Ying J, Peng S, He Q, Yang H, Yang C, Romeo M, Visperas R, Kenneth G, Cassman K (1998). Comparison of high-yield rice in tropical and subtropical environments. I. determinants of grain and dry matter yields. Field Crop Res.

[CR43] Zeng D, Tian Z, Rao Y, Dong G, Yang Y, Huang L, Leng Y, Xu J, Sun C, Zhang G, Hu J, Zhu L, Gao Z, Hu X, Guo L, Xiong G, Wang Y, Li J, Qian Q (2017). Rational design of high-yield and superior-quality rice. Nat Plants.

[CR44] Zeng M, Yang J, Wu K, Wang H, Sun K, Chen Z, Guo T, Chen C (2021). Genome-wide association study reveals early seedling vigour-associated quantitative trait loci in indica rice. Euphytica.

[CR48] Zhang Z, Yu S, Yu T, Huang Z, Zhu Y (2005). Mapping quantitative trait loci (QTLs) for seedling-vigor using recombinant inbred lines of rice (*Oryza sativa* L). Field crop res.

[CR46] Zhang F, Jiang Y, Yu S, Ali J, Paterson AH, Khush GS, Xu J, Gao Y, Fu B, Lafitte R, Li Z (2013). Three genetic systems controlling growth, development and productivity of rice (*Oryza sativa* L.): a reevaluation of the ‘Green Revolution’. Theor Appl Genet.

[CR45] Zhang A, Liu C, Chen G, Hong K, Gao Y, Tian P, Peng Y, Zhang B, Ruan B, Jiang H, Guo L, Qian Q, Gao Z (2017). Genetic analysis for rice seedling vigor and fine mapping of a major QTL *qSSL1b* for seedling shoot length. Breed sci.

[CR47] Zhang L, Huang J, Wang Y, Xu R, Yang Z, Zhao Z, Liu S, Tian Y, Zheng X, Li F, Wang J, Song Y, Li J, Cui Y, Zhang L, Chen Y, Lan J, Qiao W, Yang Q (2020). Identification and genetic analysis of *qCL1.2*, a novel allele of the green revolution gene *SD1* from wild rice (*Oryza rufipogon*) that enhances plant height. BMC Genet.

[CR49] Zhao Y, Jiang C, Rehman R, Zhang H, Li J, Li Z (2019). Genetic analysis of roots and shoots in rice seedling by association mapping. Genes Genom.

[CR50] Zhou L, Wang J, Yi Q, Wang Y, Zhu Y, Zhang R (2007). Quantitative trait loci for seedling vigor in rice under field conditions. Field Crop res.

